# Patients with persistent medically unexplained physical symptoms: a descriptive study from Norwegian general practice

**DOI:** 10.1186/1471-2296-15-107

**Published:** 2014-05-29

**Authors:** Aase Aamland, Kirsti Malterud, Erik L Werner

**Affiliations:** 1 Research Unit for General Practice, Uni Research Health Bergen, Kalfarveien 31, N-5018 Bergen, Norway; 2 Department of Global Public Health and Primary Care, University of Bergen, Bergen, Norway; 3 Research Unit for General Practice in Copenhagen, Copenhagen, Denmark

**Keywords:** Prevalence, Cross-sectional studies, Somatoform disorders, Employment, Primary health care

## Abstract

**Background:**

Further research on effective interventions for patients with peristent Medically Unexplained Physical Symptoms (MUPS) in general practice is needed. Prevalence estimates of such patients are conflicting, and other descriptive knowledge is needed for development and evaluation of effective future interventions. In this study, we aimed to estimate the consultation prevalence of patients with persistent MUPS in general practice, including patients’ characteristics and symptom pattern, employment status and use of social benefits, and the general practitioners’ (GPs) management strategy.

**Method:**

During a four-week period the participating Norwegian GPs (n = 84) registered all consultations with patients who met a strict definition of MUPS (>3 months duration and function loss), using a questionnaire with simple tick-off questions. Analyses were performed with descriptive statistics for all variables and split analysis on gender and age.

**Results:**

The GPs registered 526 patients among their total of 17 688 consultations, giving a consultation prevalence of persistent MUPS of 3%. The mean age of patients was 46 years, and 399 (76%) were women. The most frequent group of symptoms was musculoskeletal problems, followed by asthenia/fatigue. There was no significant gender difference in symptom pattern. Almost half of the patients were currently working (45%), significantly more men. The major GP management strategy was supportive counseling.

**Conclusion:**

A consultation prevalence rate of 3% implies that patients with persistent MUPS are common in general practice. Our study disclosed heterogeneity among the patients such as differences in employment status, which emphasizes the importance of personalized focus rather than unsubstantiated stereotyping of “MUPS patients” as a group.

## Background

Medically Unexplained Physical Symptoms (MUPS) captures conditions characterized by symptoms without corresponding objective findings [1], such as asthenia, low back pain, fibromyalgia, irritable bowel syndrome, or chronic fatigue syndrome. Drawing upon our clinical experience, we distinguish between everyday and self-limiting complaints as compared to *persistent* MUPS, with long-lasting symptom and loss of function.

Although the term is debated [2-4], we find its use appropriate for clinical and research purposes. First, there are substantial similarities and co-morbidity across different MUPS disorders [5,6], indicating possible common underlying symptom mechanisms [7]. Second, several studies claim similar treatment to be effective across different MUPS disorders [8]. Third, patients with MUPS have been associated with high costs, both direct (health care use) and indirect costs (productivity loss due to sickness absence) [9].

The research literature on MUPS prevalence in general practice is conflicting, with prevalence rates ranging from 1.1 to 33% [1,10-17]. This is probably due to different or imprecise definitions of the condition, or different data sources for measurement. An often-reported figure is 15% [18], while Steinbrecher et al. report more than two-thirds of all consultations to be related to MUPS [19]. A recent Cochrane review calls for more knowledge about the use of social benefits among patient with MUPS [20]. Consultation prevalence, or the proportion of consultations with a certain condition, is an adequate measure for health care utilization related to different health problems in general practice, for instance when estimating direct costs of MUPS [9].

Effective interventions for patients with persistent MUPS in general practice are yet to be established, but should be based on treatment studies where more descriptive knowledge of the patients is included. We therefore conducted a study in a naturalistic GP-setting to estimate the consultation prevalence and describe symptom pattern of patients with persistent MUPS in Norwegian general practice. We also wanted to describe these patients’ employment status and use of social benefits, and the GPs management strategy.

## Method

In planning, implementation and reporting we used the STROBE check list for cross-sectional studies [21].

### Study setting and design

Norway has 5, 05 million inhabitants and 4 189 GPs. A list system includes 99.5% of the population and connects every Norwegian citizen to a specific GP. The GPs issue 80% of all sickness certificates [22]. Health care is mostly publicly financed, with a fixed amount of maximum personal charge per year for the patient. Vest-Agder county has approximately 180 000 inhabitants and 160 GPs located in 44 different practices. It has a mix of rural and urban areas, and covers a range of socioeconomic status among the inhabitants.

In October 2012 all GPs in this county were invited by postal letter to participate in our multi-practice cross-sectional study, after piloting in a practice with 13 GPs. The GPs were asked to register all patients who met a strict definition of persistent MUPS (see below) attending during a four-week period. We defined the *consultation prevalence* as the number of consultations with patients with MUPS related to the total number of consultations across the study period. The GPs received invitation-letter and then reminder e-mails before, right after study-start and midway in the registration period. AA promoted the study through meetings with the GPs, either during lunch-visits or in supervised peer groups. One week after the registration period, all non- responders were contacted with a reminder letter, e-mail, SMS or telephone.

### Case definition and questionnaire

For the purpose of this study, we defined *persistent MUPS* as “physical symptoms with no identified organic cause, lasting for at least three months and leading to a loss of function”. *Loss of function* was explained as sickness absence or disability, or withdrawal from social activities like sports, social events or other leisure activities. In case of co-morbid diseases, the unexplained symptoms should outweigh function loss caused by medically explained symptoms. Our case definition was carefully presented in the invitation letter, e-mails and personal meetings with the GPs. Patients ≥ 18 years were included.

The GPs were asked to register data from every eligible patient on a one-page form with simple tick-off questions during a four-week period. The registration form, which was completed in less than three minutes (see Additional file 1), included relevant demographic information, symptom pattern (duration and localization), work participation and the GPs management strategy in the actual consultation.

### Statistics

The data were analyzed using IBM SPSS version 20 for Windows with descriptive statistics for all variables and split analysis on gender and age. We used independent-samples-t-tests for comparing means on continuous variables for two different groups of participants, and Chi-square test for independence to explore the relationship between categorical variables. Possible relationship between number of registered patients and different GP-characteristics were investigated using simple bivariate correlation. The level of statistical significance was set to p < 0.05.

## Results

### Participating GPs

The response rate was 53% (84/160). The invited GPs were representative for Norwegian GPs, although with an excess of men and GP specialists (Table 1). Participating male GPs were significantly older (p = 0.011), and had worked more years in general practice than the females (p = 0.002) (Table 2). There was a variation in the number of recorded consultations with patients with persistent MUPS (MUPS consultations) per GP (range 0–26, mean = 6.38, SD = 4.97). No significant associations were found between number of recorded MUPS consultations and gender, age, specialist status, years in practice, or the total number of consultations during the registration period among the GPs (Table 2).

**Table 1:** Characteristics of all Norwegian GPs, GPs in Vest-Agder and the participating GPs

	**Norway**	**Vest-Agder**	**Participants**
	**n = 4 189**	**n = 160**	**n = 84**
***Age, mean***	48.5 y	48.1 y	48.0 y
***Women***	36.5%	33.5%	29.8%
***Mean number of patients listed per doctor***	1 164	1 083	1 107
***Specialty attainment***	55.0%	62.0%	72.6%

**Table 2:** Demographics of the participating GPs (n = 84; men 59, women 25), and their proportion of patients with persistent MUPS among all consultations during the four-week registration period

	**Total**			**Men**			**Women**		
	**Mean**	**(SD)**	**Range**	**Mean**	**(SD)**	**Range**	**Mean**	**(SD)**	**Range**
Age*	48.0	(11.37)	26-68	49.9	(11.63)	26-68	43.5	(9.49)	26-60
Years in practice*	17.8	(12.15)	0-42	20.3	(12.27)	0-42	11.8	(9.98)	0-32
***List length***	1107	(340.0)	370-2496	1126	(364.2)	370-2496	1061	(277.4)	460-1450
***MUPS-patientsˡ***	6.38	(4.97)	0-26	6.32	(5.39)	0-26	6.52	(3.91)	0-15
***N consultations***	210.33	(82.55)	48-394	218.8	(77.8)	57-363	190.4	(91.40)	48-394

*Difference between genders is significant at the 0.05 level.

ˡAssociation between number of patients with persistent MUPS and total consultations at the level of 0.01 (pearson p = 0.001, spearman, p = 0.003).

### Descriptive data and demographics of the registered patients

The GPs registrated 526 MUPS consultations out of a total of 17 688 consultations, which gave a consultation prevalence rate of 3%. Of these were 399 (75.9%) with female patients. The educational level among the patients did not differ from the distribution in the population in the county as a whole [23]. 234 (44.7%) of the patients were working, significantly more men (p = 0.047) (Table 3). Among the 289 not working, 91 (31.5%) were on sick leave, 119 (41.2%) had a disability pension, and 76 (26.3%) had other social benefits, mostly work assessment allowance. There was no significant gender difference in patients’ age, education level or duration of symptoms (Table 4), or symptom pattern (Table 3). Musculoskeletal pain and asthenia/fatigue were predominant symptoms, with 68.1% and 57.0% of all patients respectively. Headache or dizziness was reported by 31.9% of the patients and gastrointestinal symptoms by 20.5%.

**Table 3:** Patients with persistent MUPS (n = 526, men 125, women 399)

	**All**		**Men**		**Women**	
**Symptom pattern, n (%)**						
Gastrointestinal	108	(20.5)	26	(20.5)	82	(20.6)
Musculoskeletal	358	(68.1)	78	(61.4)	280	(70.2)
Headache/dizziness	168	(31.9)	36	(28.3)	132	(33.1)
Asthenia/fatigue	300	(57.0)	72	(56.7)	228	(57.1)
Others	41	(7.8)	10	(7.9)	31	(7.8)
**Work status, n (%)**						
Working *	234	(44.7)	67	(52.8)	167	(42.2)
**Not working**						
Sick leave	91	(31.5)	18	(30.0)	73	(31.9)
Disability pension	119	(41.2)	25	(41.7)	94	(41.0)
Other	76	(26.3)	18	(30.0)	58	(25.3)
**GPs management, n (%)**						
Supportive counceling	333	(63.7)	83	(65.4)	250	(63.1)
Physical examination	195	(32.3)	50	(31.4)	145	(36.6)
Prescriptions	124	(23.7)	27	(21.3)	97	(24.5)
Blood test	113	(21.6)	20	(15.7)	93	(23.5)
Referral	93	(17.8)	24	(18.9)	69	(17.4)
Social security certificates	106	(20.3)	25	(19.7)	81	(20.5)
Others	41	(7.8)	11	(8.7)	30	(7.6)

Symptom localization, work status and GPs management in numbers and percent.

*Difference between genders is significant at the 0.05 level.

**Table 4:** Characteristics of patients with persistent MUPS (n = 526) in numbers and percent

	**All**		**Men**		**Women**	
**Gender**			127	(24.1%)	399	(75.9%)
**Age (years)**						
Mean	46.11	(SD = 13.92)	45.74	(SD = 14.09)	46.23	(SD = 13.88)
18-40	182	(35.0)	43	(34.7)	139	(35.1)
41-65	291	(56.0)	70	(56.6)	221	(55.8)
>65	47	(9.0)	11	(8.7)	36	(9.1)
**Education (n, %)**						
Primary school	131	(25.5)	30	(24.4)	101	(25.9)
Highschool	249	(48.6)	67	(54.5)	182	(46.7)
College/university	133	(25.9)	26	(21.1)	107	(27.4)
**Duration of MUPS (n, %)**						
<1 year	73	(14.0)	25	(20.0)	48	(12.1)
1-5 years	161	(30.9)	39	(31.2)	122	(30.8)
> 5 years	287	(55.1)	61	(48.8)	226	(57.1)

Age, level of education and symptom duration.

### GPs management

The majority of the GPs reported that they had offered supportive counseling in the actual MUPS consultations*.* Medication was prescribed in 21.6% of the consultations. About 20% of the consultations included lab tests or referrals. There were no significant gender differences in GPs management strategy (Table 3).

## Discussion

### Summary

This study presents for the first time cross-sectional descriptive data on consultations with patients with persistent MUPS in Norwegian general practice. A consultation prevalence rate of 3% suggests that patients with persistent MUPS are common when compared to other reasons for encounter. Musculoskeletal symptoms were the most frequent symptom pattern, followed by asthenia/fatigue. Almost 45% of the patients were working. About 2/3 of the GPs offered supportive counseling at the actual consultation.

### Strengths and limitations

Our study is strengthened by the naturalistic setting and the rigorous case definition, which was carefully described, also through a clinical vignette during personal meetings. Our case definition resembles definitions used in previous research [12,13,15]. The prevalence in this study is on MUPS patients as perceived by Norwegian GPs and not a prevalence of MUPS in the general public, and due to the study design one patient may have been registered several times, which may influence the results. We believe however this not to be very likely and does not affect the prevalence rate, as this is still a prevalence of MUPS consultations in general practice.

Our design did not allow for inter-rater-reliability checks of the GPs’ assessments. As mean duration of a GP-patient relationship in Norway is 7.7 years [24], we may assume GPs to easily recognize eligible patients due to their knowledge about their patients. Data collection based on digital patient records or interviews may represent larger sources of bias, due to lack of a specific MUPS-diagnosis, incomplete medical records or interviewer’s lack of personal knowledge of the actual patients.

Sample bias was reduced through an overall representative sample of GPs-, indicating that the results may be generalised to a comparable setting. However, more participating male GPs may underestimate the consultation prevalence since female GPs see more female patients, and, due to gender effects on communication, they may see more patients with MUPS [25]. GP based prevalence estimates are found to be consequently underestimated [15,26], but this may also indicate registration of symptoms of less clinical significance, which we did not intend to include in this study.

A response rate of 53% is modest, although higher than other surveys of GPs on MUPS [27,28]. Furthermore, our sample seemed to be fairly representative for Norwegian GPs.

Possible source of information bias may be due to incomplete recordings due to GPs busy workday [29,30] resulting in ignored eligible patients (consultation prevalence underestimated), or a non-compliant attitude to the actual instruction due to an eager to participate because of professional relationships with the first author (skewing possible for both directions). The GPs in the area received various reminders and information about the study. How this may have influenced the registration is unknown [31]. On the other hand, personal and extensive study information may have raised the actual quality of the collected data.

Only 32% of the patients in our sample were on sick leave. This finding seems to oppose previous research [32] by suggesting that previous assumptions of high level of disability among patients with persistent MUPS needs revision. However, as loss of function was a criterion for eligibility in the study, we can only assume that that these patients met this criterion otherwise than being out of work.

### Comparison with existing literature

A consultation prevalence rate of 3% suggests that patients with persistent MUPS are common in general practice when compared to other frequently occurring conditions like hypertension, atrial fibrillation, diabetes mellitus, and depression, each accounting for 1.3-4.9% of the consultations in Norwegian general practice [33]. A recent retrospective study from primary and secondary care in US [12] presented exactly the same consultation prevalence for persistent MUPS by use of a similar case definition, and a Dutch study from primary care found a prevalence of 2.45% on persistent MUPS [11].

More women (76%) than men in our sample suffered from MUPS, consistent with prior studies [34]. However, we should keep in mind that doctors tend to categorize female patients more easily as somatizers than male ones [35] and that this may be the same for patients with MUPS as we do not see persistent MUPS as synonymous with ‘severe somatization disorder’ [36].

In contrast to findings from a similar study we found linearity between number of patients with persistent MUPS and total number of consultations at the individual GPs [12]. Musculoskeletal symptoms were the most frequent MUPS symptom pattern in our study, followed by asthenia/fatigue. These findings diverge from a recent study where asthenia/fatigue was leading among patients with persistent MUPS [34]. However use of different case definitions may contribute to this difference, and with knowledge of substantial co-occurrence for these symptoms, this finding may be of less clinical importance.

An important overall finding challenges the stereotypes of patients with persistent MUPS. In our study, this group of patients is heterogenous - a quarter are men, a quarter has higher education, a third is below age 40, and nearly half of them work. This highlights the need to refrain from unsubstantiated stereotyping of “MUPS-patients”.

Our finding of a scarce prescription rate complies with recommendations that medication should play a minor role in medical management of MUPS [37,38]. Our case definition implied prior appropriate diagnostic evaluations, but still we found that one out of five patients received further referrals. This may illustrate GPs feeling of shortcoming in managing these patients, and a lack of knowledge of what these patients’ actual want and need [39,40]. Furthermore, it may illustrate a limited awareness among the GPs about potentially iatrogenic harms trough additional interventions [41]. However, actual lab test and referrals in the registered MUPS consultations may represent adequate management of other co-existing diseases or presentation of new symptoms, which may represent new disease.

Levels of supportive counseling at the GP encounter will probably vary according to structure of health care system. In a study from US, 37% were referred to different mental health care center [12], reflecting possible differences in therapeutic cultures. It seems like Norwegians GPs more often provide supportive care themselves rather referring to secondary care.

## Conclusion

This study reveals that consultations with patients with persistent MUPS are common in general practice. These consultations represent a heterogeneous group of patients, which illustrate possible needs for modifications of stereotyping upon these patients. A continuous focus on GPs knowledge and management skills of MUPS is important, as GPs have a major role in caring for these patients. More research on effective interventions, based on knowledge about the heterogeneity of this group of patients, is needed.

### Additional information

#### Ethical approval

Only the individual GP knew the identity of the patients and all personal data were anonymous. Therefore, neither The Regional Committee for Ethics in Medical Research nor the Norwegian Social Science Data Services (NSD) regarded the study to be within their mandatory (Reference number 31527/3/AMS).

### Keypoint

• Patients with persistent MUPS account for 3% of consultations in Norwegian general practice;

• Musculoskeletal symptoms were most frequent, followed by asthenia/fatigue;

• 45% of patients with persistent MUPS were currently working, of these significantly more men;

• A major GP-management strategy was supportive counseling. GPs need tools to identify and provide adequate health care for patients with persistent MUPS.

## Competing interests

The authors report no competing interest. The authors alone are responsible for the content and writing of the paper.

## Authors’ contributions

All authors designed the study. AA conducted the data collection, analysis and led the drafting of the manuscript. ELW and KM contributed to analysis, discussion of findings and drafting of the manuscript. All authors read and approved the final manuscript.

## Pre-publication history

The pre-publication history for this paper can be accessed here:

http://www.biomedcentral.com/1471-2296/15/107/prepub

## Supplementary Material

Additional file 1 Registration form. Click here for file
