# Supplementary material for: Patients with persistent medically unexplained physical symptoms: a descriptive study from Norwegian general practice
Source: BMC Fam Pract. 2014 May 29;15:107. doi: 10.1186/1471-2296-15-107 (PMC4041895; doi:10.1186/1471-2296-15-107)
Supplement: Additional file 1 — Registration form. [file 1471-2296-15-107-S1.docx]

*This patient has ”subjective symptoms” without corresponding ”objective” findings after investigation. The symptoms have lasted for at least three months.*

**Demographics of the patient:**

Gender: ⬜_1_ male ⬜_2_ female Year of birth:

Education level:

⬜_1_ Primary school ⬜_2_ High school ⬜_3_ College/university

Duration of MUPS:

⬜_1_ <1 year ⬜_2_ 1-5 years ⬜_3_ 5-10 years

**Question 1: Where do the patient localize his/her symptoms? Several options possible**

⬜_1_ Gastrointestinal system

⬜_2_ Musculoskeletal system

⬜_3_ Headache / dizziness

⬜_4_ A general feeling of asthenia or fatigue

⬜_5_ Other_________________________

**Question 2: Is the patient working?** ⬜_1_ yes ⬜_2_ no

If no:

⬜_1_ Sick listed from today

⬜_2_ Allready sick listed due to MUPS

⬜_3_ Allready sick listed due to other reasons than MUPS

⬜_4_ Disability pension

⬜_5_ Pensioner /student/home-working (not benefit rights)

⬜_6_ Return to work today

⬜_7_ Other

**Question 3: Content of the consultation (related to the symptoms in q. 1): Several options possible**

⬜_1_ Supportive counceling

⬜_2_ Physical examination

⬜_3_ Prescriptions, if yes, what kinds? ______________________________________⬜_4_ Blood test(s)

⬜_5_ Referral(s) (all types)

⬜_6_ Social security attestations

⬜_6_ Others: ____________________________
